# Remedy for Tape Worm

**Published:** 1853-05

**Authors:** J. M’Creary Sudduth

**Affiliations:** Indian Point, Menard Co., Ill.


ART. II.—*Remedy for Tape Worm.* By J. M'CREARY SUDDUTH, M. D.

MESSRS. EDITORS:—I saw announced in the Sept. No. of the *North-Western Medical and Surgical Journal* that pumpkin seed and also that honey were remedies for tape-worm. An article also appeared in the Nov. No. of the *Western Journal of Medicine and Surgery* highly recommending paste made of pumpkin seed and honey, as a remedial agent in cases of Tænia. About the time that the last named article appeared, Dr. Smick and myself were consulted by a patient evidently possessing and much annoyed by one of those troublesome parasites. Wishing to give all new remedies emanating from a respectable source a fair trial, we advised him to use the above-named articles, and when next heard from he had tried the remedies, the result of which to us was so satisfactory, that I thought I would report the case to your Journal for publication.

Case B. H. male, aged 28—occupation farmer, volunteered in 1847 to go to Mexico. In good health when he left home (Ky.) Sickened while on the gulf, Nov. '47. Landed and went as far as the City of Mexico: though unfit for duty, and on the sick list during his entire stay in Mexico. Discharged and returned to Kentucky early in the summer of '48, quite weak and much emaciated. In August of '48 began to pass by stool small portions of what proved afterwards to be joints of tape worm. Shortly after his return to Kentucky, he applied to a number of Physicians for advice; receiving no benefit from any article recommended, except those used by one doctor whose prescription brought away eight feet of tape worm. Patient thinks the articles used by him were cal. and Dr. Jayne's vermifuge. Shortly after passing this piece of worm, finding himself not relieved, and knowing now what it was that troubled him, he went to Louisville and consulted a number of physicians of that place, offering one hundred dollars to any one who would rid him of his troublesome companion. He however received no benefit from any articles used by physicians of that place. Soon after leaving Louisville, he came to Illinois; still poor and in bad health. He consulted with all the doctors that came in his way, using all articles by them recommended, as well as all the patent medicines that he saw extolled for the removal of tape worm, (and their name is legion) in newspapers, almanacs and receipt books, &c.; however all failed. When he consulted Dr. Smick and myself, he presented the appearance and symptoms as follows: Was lean in flesh, and æmenic, judging from the appearance of *the surface* and color of his lips. Abdomen somewhat distended, troubled much and especially at night by a moving, creeping sensation in the abdomen. Variable appetite, at times voracious, at others none at all, passing daily by stool a number of joints of the worm. We advised him to eat of the paste of pumpkin seed and honey, ℥iij, one ounce at a time, with an interval of two hours. Six hours after taking the last portion he passed twenty two feet of the worm, though in three pieces, the longest of which was eighteen feet, and bearing the head of the parasite. I have been thus particular in discribing the above case, to show the difficulty attending the expulsion of this parasite by the usual articles prescribed by physicians, for this purpose; showing the superiority of paste of pumkin seed and honey over all known articles for the removal of this troublesome if not destructive parasite. The worm removed belonged to that class of tape worm technically called Tænia Solium. Since the removal of the worm, the patient has complained, after eating, of a sick disagreeable sensation in the region of the stomach, his health otherwise much improved. All symptoms of worms have disappeared.

Indian Point, Menard Co., Ill., March 14, 1853.

